# Melissopalynological and Physicochemical Analysis of Selected Honey Samples from Romania, Spain, Malaysia, and New Zealand

**DOI:** 10.3390/foods15091517

**Published:** 2026-04-27

**Authors:** Corina-Bianca Ioniță-Mîndrican, Robert Ancuceanu, Mihaela Dinu, Eliza Oprea, Carolina Negrei

**Affiliations:** 1 Department of Toxicology, Faculty of Pharmacy, “Carol Davila” University of Medicine and Pharmacy, 020956 Bucharest, Romania; corina-bianca.ionita-mindrican@drd.umfcd.ro (C.-B.I.-M.); carolina.negrei@umfcd.ro (C.N.); 2 Department of Pharmaceutical Botany and Cell Biology, Faculty of Pharmacy, “Carol Davila” University of Medicine and Pharmacy, 020956 Bucharest, Romania; mihaela.dinu@umfcd.ro; 3 Department of Botany and Microbiology, Faculty of Biology, University of Bucharest, 77206 Bucharest, Romania; eliza.oprea@g.unibuc.ro

**Keywords:** honey, pollen, melissopalynology, monofloral, multifloral

## Abstract

Melissopalynological analysis (the microscopic examination of pollen in honey) provides valuable information regarding both the geographic and botanical origins of honey. This study aims to verify the authenticity of different types of honey by assessing their claimed floral sources and, indirectly, locations. Twelve samples of various botanical origins were collected: from Romania (linden, black locust, rapeseed, hawthorn, mint, thyme, multifloral, pasture, manna), New Zealand (Manuka honey), Spain (chestnut honey), and Malaysia (Tualang honey). In the study, 16 botanical families were identified across the 12 types of honey analyzed. The frequency of botanical families shows that Fabaceae, Asteraceae, and Brassicaceae are the most widespread. A moderate frequency was observed for Apiaceae, Lamiaceae, and Poaceae. Other families had a lower distribution, such as Myrtaceae and Ericaceae. Rapeseed honey was the most abundant monofloral honey type based on species level pollen dominance (96.86%), followed by chestnut honey (94.17%) and linden honey (84.45%). Meanwhile, thyme honey (52.84%) and mint honey (51.1%) had a specific pollen abundance just over 50% at the family level (Lamiaceae).

## 1. Introduction

Determining the authenticity of honey is a very important step for ensuring the quality and purity of the product, protecting consumers from possible adulterations, and increasing their level of trust. Honey is a sweetener that has been used since ancient times. In addition to its high sweetening power, numerous studies have demonstrated many benefits that consumers can gain from using honey [1,2]. For example, the specialized literature has shown its antioxidant, antibacterial, and anti-inflammatory effects [3,4,5]. To detect honey adulteration, several laboratory analyses are available, such as characterization based on their physicochemical composition or based on melissopalynological analysis (i.e., the study of pollen grains present in honey through microscopic examination) [2].

Honey has a complex composition with a high content of carbohydrates (80–85%) and water (15–17%) [6], as well as compounds such as phenolic acids [7], flavonoids, vitamins, and minerals [8,9,10,11]. On the other hand, honey can also contain compounds resulting from contamination. Primary sources include pollen and dust, while secondary sources can be generated during the honey handling process. Besides the differences that may occur between various types of honey depending on their physicochemical properties, honey can also be differentiated based on the geographical area, which is reflected in the local flora. This leads to a different distribution of botanical families depending on the region. Thus, melissopalynological analysis of pollen from plants belonging to different botanical families plays a fundamental role in the study of honey [1].

**Figure 1:**
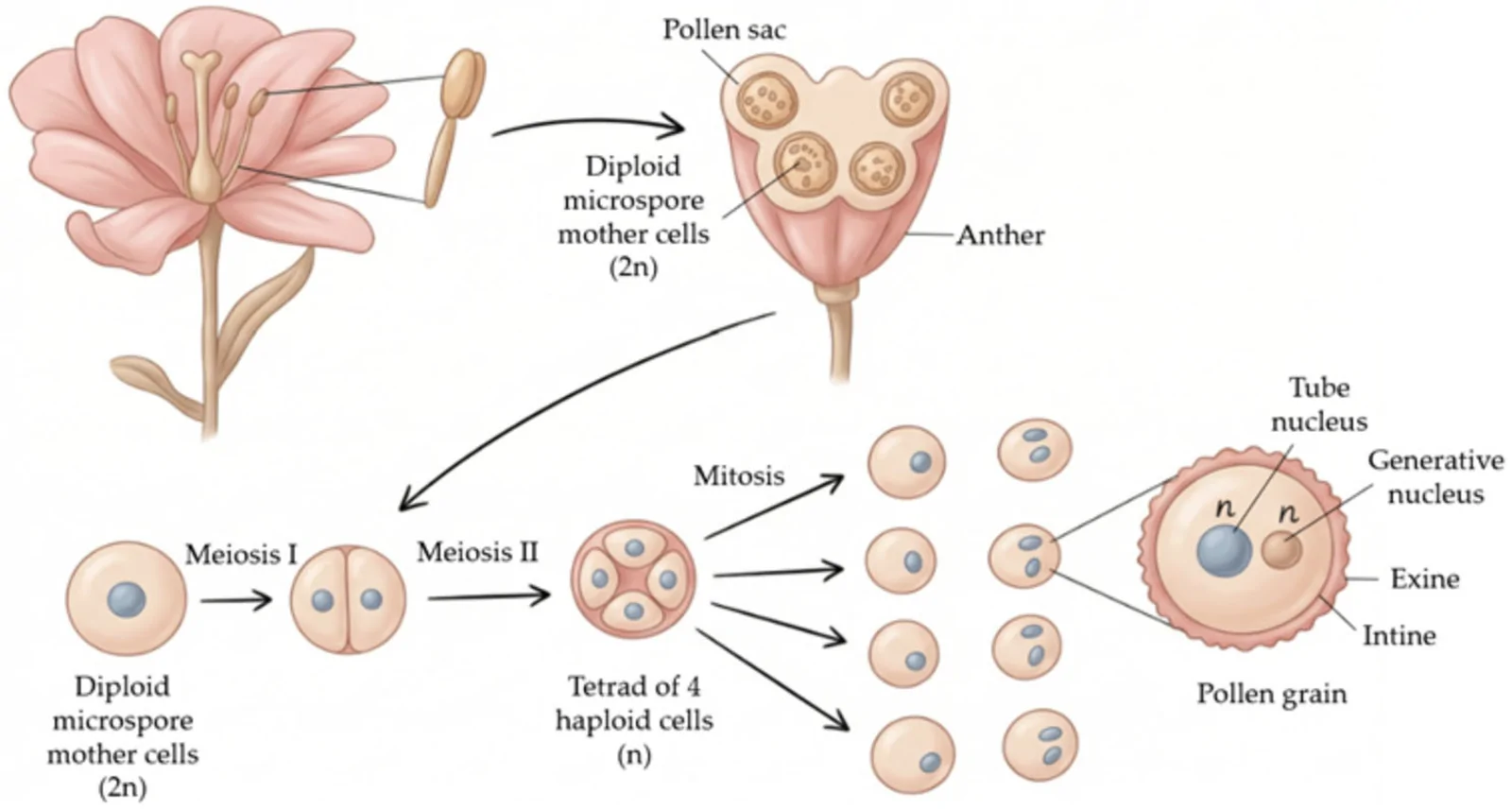
Pollen development in angiosperms.

Melissopalynological analysis relies on understanding the pollen grain formation (Figure 1), as structural characteristics such as exine (smooth, reticulate, or echinate), pollen size, and aperture type are essential for microscopic identification [12].

Melissopalynology is used for the botanical determination of honey because each pollen grain has a structural pattern specific to each floral species or supraspecific taxon. In addition to identifying the type of honey, melissopalynology also provides information about the plant sources of pollen and nectar used by honeybees in the geographic region for honey production. During the analysis, it can be determined if the honey is monofloral or multifloral. Monofloral honey is a type in which the pollen of a single species predominates, whereas in multifloral honey, the proportions of pollen grains come from multiple botanical species without a marked predominance of any single one [1].

Monofloral honey does not contain exclusively a single type of pollen, but rather a predominance of pollen from a particular floral plant source, collected from a specific plant species [13]. Some plants can provide more pollen while others provide less nectar; thus, the pollen from certain floral products may be overrepresented, as is the case with *Brassica* sp. pollen, while others may be underrepresented [14].

Pollen analysis can be used as a method for authenticity control. In honey, this analysis provides important information about the extraction and filtration, possible adulteration, and hygienic quality. According to studies, for accurate results, a count of at least 300 pollen grains is generally recommended for general orientation and between 500 and 1000 grains for precise measurements. For determining the botanical origin, it is necessary to count between 500 and 1000 pollen grains. Nevertheless, pollen can come both from the primary floral source, through nectar from flowers collected by bees, and from secondary, tertiary, and quaternary sources. Therefore, melissopalynological analysis is carried out with great caution. Secondary enrichment refers to pollen originating from the hive, tertiary enrichment may occur during the honey extraction process, whereas quaternary enrichment results from airborne contamination. Honey is considered monofloral if the relative frequency of pollen from that taxon exceeds 45% [14].

## 2. Materials and Methods

### 2.1. Honey Samples and Geographical Origin

Melissopalynological analysis was carried out on honey samples originating from Romania (n = 9), New Zealand (n = 1), Spain (n = 1), and Malaysia (n = 1). The honey samples were collected both from beekeepers and from supermarkets from trusted brands. Honey samples were selected to cover a diverse range of botanical and geographical origins. Both European and non-European samples were analyzed, enabling a broader assessment of the pollen spectrum across different regions. The honey samples were obtained from both beekeepers and commercial sources (supermarkets), reflecting typical market availability. Commercial samples, including Manuka honey (New Zealand), Tualang honey (Malaysia), and chestnut honey (Spain), were purchased from supermarkets, as these products were not available directly from local beekeepers. The presentation of the honey samples regarding their source and geographical origin is shown in Table 1. All honey samples were stored in a dark place at room temperature prior to analysis.

Romania is located in southeastern Europe. The climate is temperate-continental, with an average annual temperature ranging from 11 to 12 °C in the southern part of the country to 0–2.7 °C in mountainous areas, with a frost-free period of 200–225 days in the south and 150 days in the mountains. Relative humidity is particularly high in winter (>80%) and low in summer (periods of “dry days” in the eastern part of the country). These environmental characteristics particularly favor the growth of plants adapted to heat and drought [15].

New Zealand is characterized by a temperate oceanic climate favorable to the growth of species from the Myrtaceae family, the main plant source of Manuka honey [16,17]. Situated in southwestern Europe, Spain has a typical Mediterranean climate featuring dry, warm summers and relatively gentle winters [18].

Malaysia is located in the equatorial region of Southeast Asia, with a tropical climate characterized by abundant rainfall and high temperatures [19].

It should be emphasized that pollen composition may vary depending on geographical region, as well as seasonal conditions.

### 2.2. Melissopalynological Analysis

Honey samples were diluted with distilled hot water (not above 40 °C) at a ratio of 1:2 (*w*/*v*), using 5 g of honey with 10 mL of distilled water and 10 g of honey with 20 mL of distilled water and centrifuged at 10,000 rpm for up to 30 min. Despite these parameters, the method failed to produce any sediment that could allow for reliable microscopic analysis. We also used three different centrifuges with no improvement. This suggested that the samples have been filtrated, and by discussion with two Romanian beekeepers this has been confirmed, as they indicated that the honey is routinely filtered to remove impurities such as dust, airborne particles, and hive debris in order to improve its clarity and esthetic appearance for consumers. Therefore, the samples were analyzed directly under the microscope. Each honey sample was analyzed using multiple independent preparations. Approximately 20–70 µL of honey was placed on a microscope slide, and the preparation was repeated as needed to ensure a sufficient number of pollen grains for analysis. When a single preparation did not provide an adequate number of pollen grains, additional preparations from the same sample were examined. The identification and quantification of pollen grains were carried out using a Nikon Labophot II (Nikon Corporation, Tokyo, Japan) light microscope (10× and 40× objectives). For each sample, a minimum of 500 pollen grains was counted (approximately 300 for low pollen honeys such as manna and pasture), in accordance with harmonized melissopalynological recommendations. Pollen identification was performed by comparison with images from the PalDat database (Palynological Database) [20].

A micrometric calibration slide was used to scale the pollen grains, which were then analyzed with ImageJ software (version 1.54, National Institutes of Health, Bethesda, MD, USA). The applied reference scale is 10 µm for all micrographs. For each type of honey, a figure was generated in which the identified pollen types and their corresponding percentages are displayed. Graphical representations for visual comparison of honey types were created in Flourish Studio (Flourish, London, UK).

### 2.3. Physicochemical Analysis

The physicochemical parameters of honey samples (moisture content, hydroxymethylfurfural (HMF), reducing sugars, apparent sucrose, and diastase activity) were determined using standardized methods in accordance with SR 784/3:2009 [21].

The moisture and hydroxymethylfurfural (HMF) content were measured by refractometry (SR 784/3:2009, point 4.1.1) and by the Winkler spectrophotometric method (SR 784/3:2009, point 4.9), respectively [21].

Reducing sugars and apparent sucrose content were determined by the Elser volumetric method, while diastase activity was assessed using the Gothe enzymatic method (SR 784/3:2009, points 4.4 and 4.5, respectively, point 4.7) [21].

## 3. Results

### 3.1. Pollen Spectrum of Individual Honey Types

Twelve honey samples were analyzed to determine the pollen spectrum.

#### 3.1.1. Linden Honey

The size of pollen from linden species ranges between 26 and 35 µm. The grains have a circular contour in polar view and feature three apertures, being tricolpate. They exhibit reticulate ornamentation [20].

**Figure 2:**
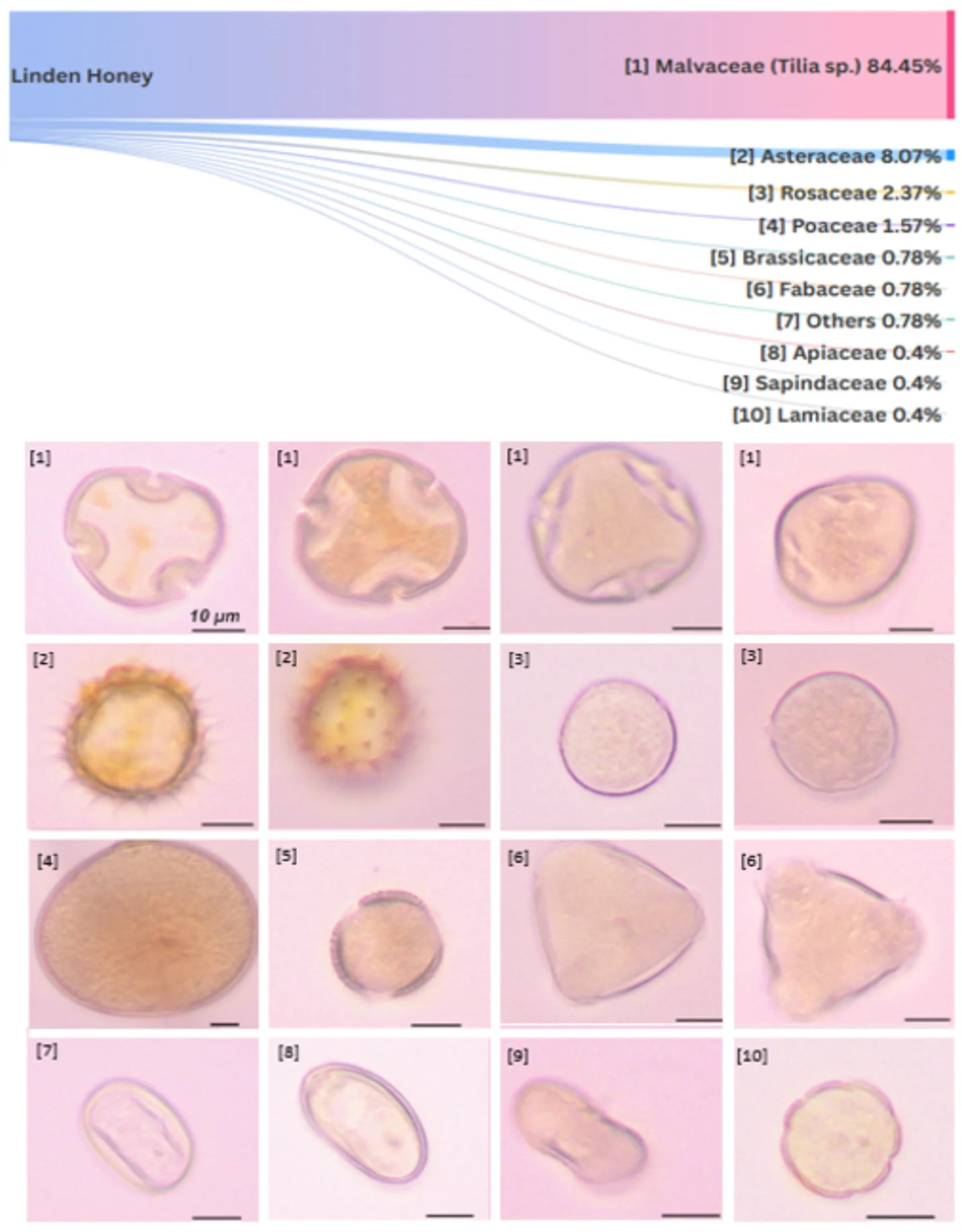
Pollen grains in linden honey [1]–[10].

In the melissopalynological analysis of linden honey, pollen grains originating from nine families were identified, while 0.78% could not be identified. The predominant family is Malvaceae, accounting for 84.45% (Figure 2). In addition to pollen from linden species, significant amounts of pollen from the Asteraceae family (8.07%) were found.

Minor pollen types were identified from Rosaceae, Brassicaceae, Poaceae, and Fabaceae (0.7–2.5%). The presence of pollen from the Poaceae family in honey may result from airborne contamination. Pollen from the Brassicaceae and Fabaceae families represents less than 1%, small quantities but which can provide information about other floral plant sources available during the harvesting period. Marginally, a percentage under 0.5% originates from plants belonging to the Apiaceae, Sapindaceae, Lamiaceae families, and other taxa (<0.5%), which have no impact on the dominant character of the assortment.

#### 3.1.2. Rapeseed Honey

Rapeseed pollen grains measure 26–30 μm and are tricolpate with reticulate ornamentation [20].

**Figure 3:**
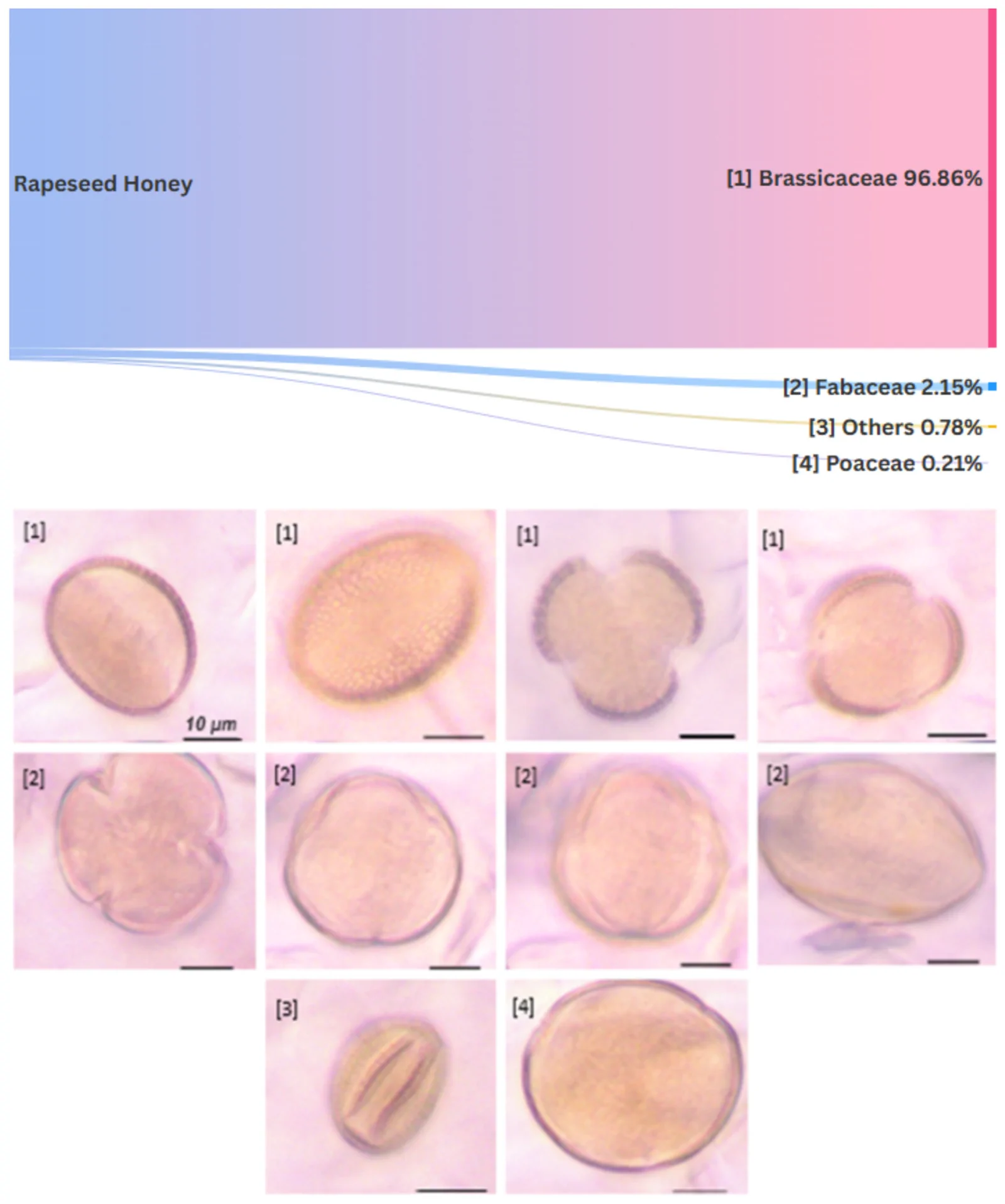
Pollen grains of rapeseed honey [1]–[4].

The analysis of rapeseed honey highlighted a structure predominantly dominated by the Brassicaceae family, which accounted for 96.86% of the total pollen grains, consistent with a monofloral pollen profile (Figure 3).

In addition to the massive presence of Brassicaceae pollen in the honey, a few secondary families were identified, such as Fabaceae (2.15%) and Poaceae (0.21%). The “Others” category accounts for 0.78% of the total pollen grains.

#### 3.1.3. Black Locust Honey

Black locust pollen appears in the form of monads with dimensions of 26–30 µm. The grains have a circular outline in polar view with three colpi (tricolpate) and psilate ornamentation with a smooth surface [20].

**Figure 4:**
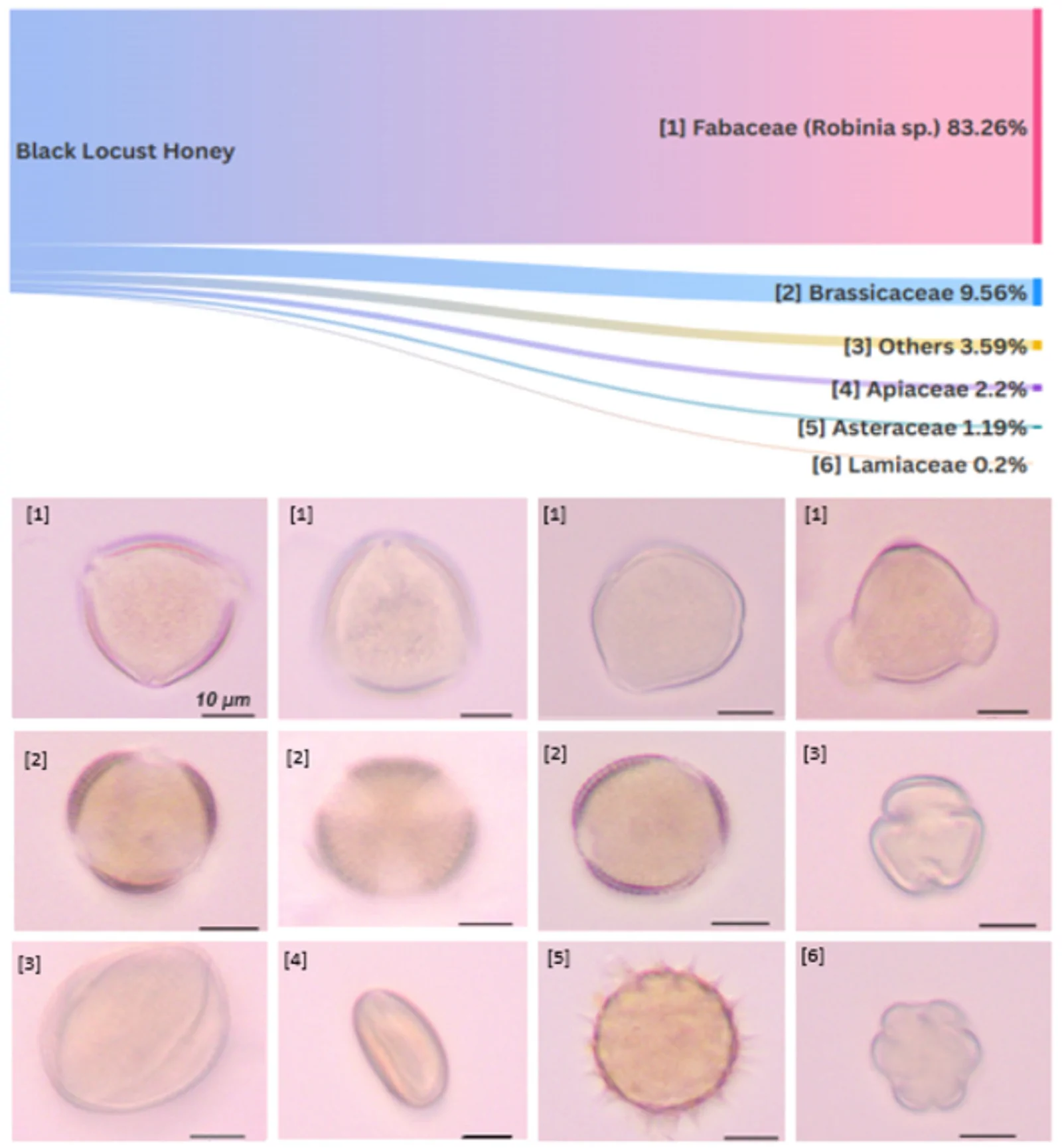
Pollen grains of black locust honey: [1]–[6].

In the analysis of black locust honey, Fabaceae pollen accounted for 83.26% of the total pollen grains (Figure 4). Secondarily, Brassicaceae pollen is found at 9.56%, followed by minor categories such as Apiaceae (2.2%), Asteraceae (1.19%), Lamiaceae (0.2%), and Others (3.59%). These accessory pollens fall within the category of secondary pollen.

#### 3.1.4. Hawthorn Honey

*Crataegus* sp. pollen grains measure 41–100 μm and are tricolporate with striate ornamentation [20].

**Figure 5:**
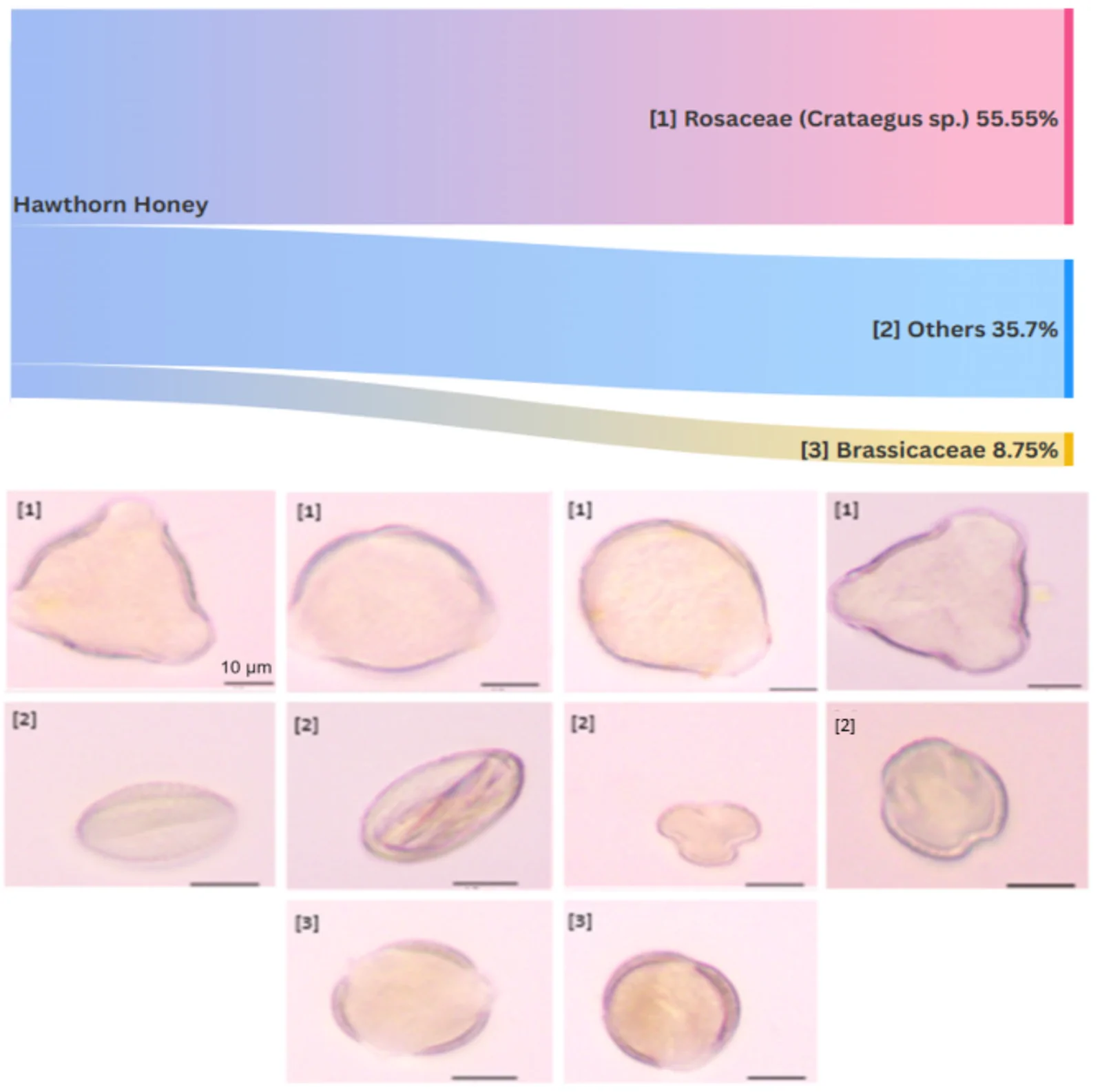
Pollen grains of hawthorn honey: [1]–[3].

For the analysis of the hawthorn honey sample, it was observed that the pollen grain count showed a predominance of 55.55% from the Rosaceae family (*Crataegus* sp.). This being slightly over 50% confirms the main botanical origin and supports the classification of the honey as monofloral hawthorn (Figure 5). Pollen from Brassicaceae represents 8.75%, indicating a secondary, but relevant, contribution of species from this family, which are known to be frequently exploited by bees during the spring period. The grains marked [3] in Figure 5 show their typical shape: spheroidal to slightly oblate, with a reticulated ornamentation, which are features corresponding to the palynological descriptions for this family. “Others” pollen that could not be precisely identified accounted for 35.7% of the total pollen grains.

The melissopalynological data reflect a floral profile dominated by hawthorn, complemented by pollen originating from plant sources.

#### 3.1.5. Mint Honey

Pollen from Lamiaceae species (*Mentha* sp. and *Thymus* sp.) measures 21–35 μm and is hexacolpate (stephanocolpate) with reticulate ornamentation [20].

**Figure 6:**
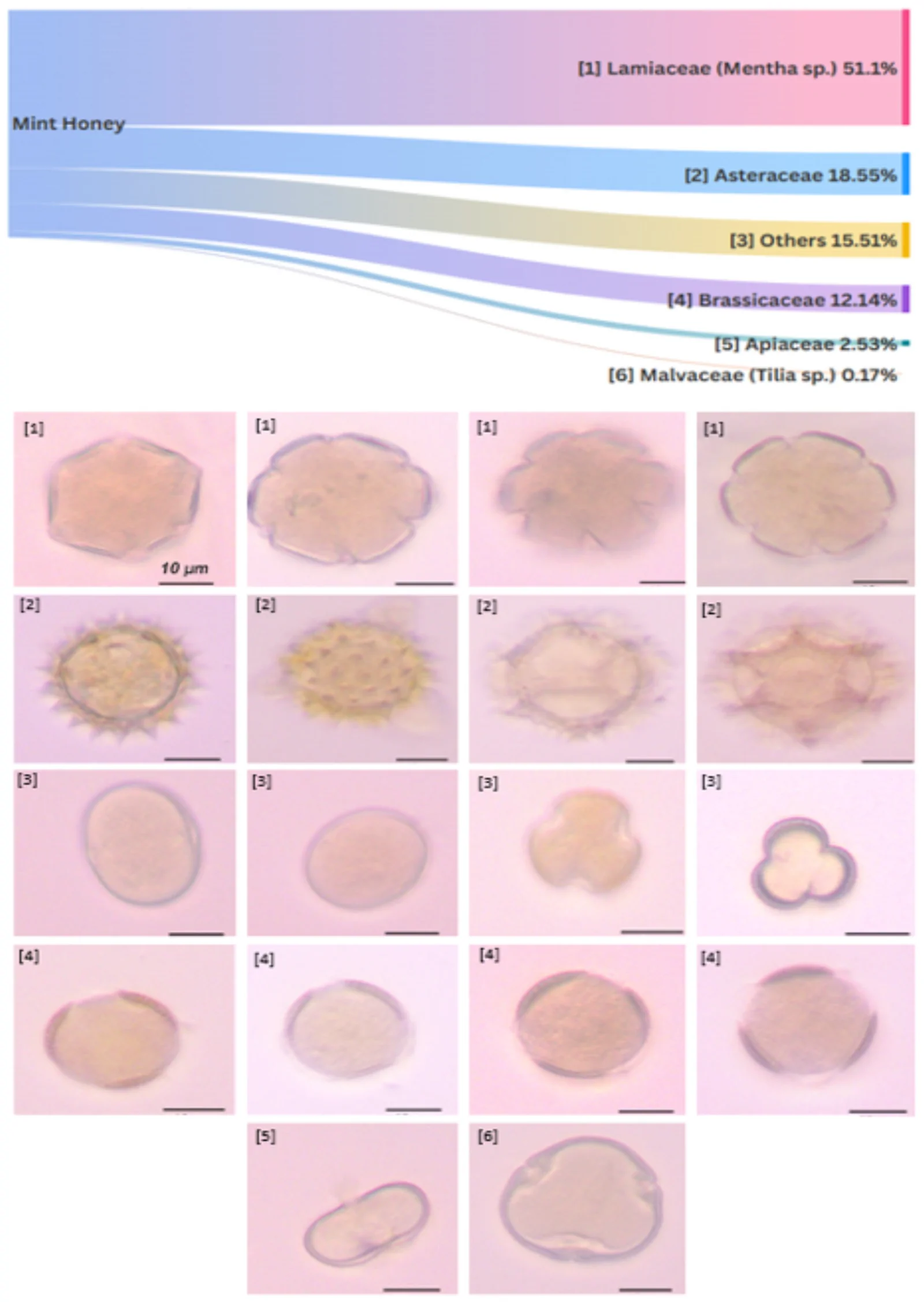
Pollen grains of mint honey: [1]–[6].

In the mint honey sample, Lamiaceae pollen (*Mentha* sp.) accounted for 51.1% of the total pollen grains (Figure 6). Secondarily, pollen from Asteraceae (18.55%) with characteristic echinate ornamentation was also identified, followed by Brassicaceae (12.14%). Additional pollen was identified from Apiaceae (2.53%), Malvaceae (0.17%), and other unidentified pollen (15.51%).

#### 3.1.6. Thyme Honey

**Figure 7:**
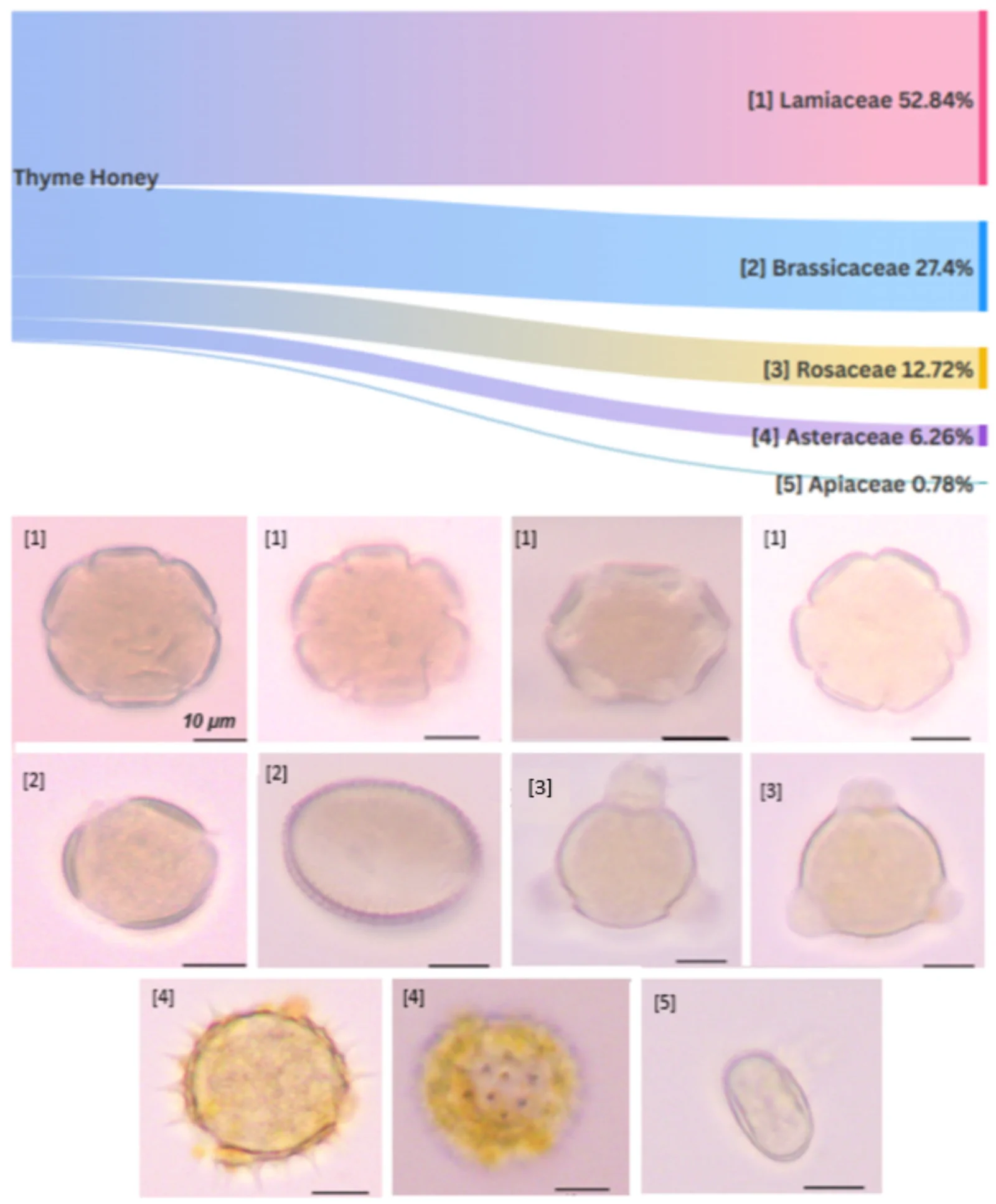
Pollen grains of thyme honey: [1]–[5].

Lamiaceae pollen constituted 52.84% of all pollen grains in the thyme honey, consistent with its botanical origin. Other identified families included Brassicaceae (27.4%), Rosaceae (12.72%), and Asteraceae (6.26%) (Figure 7).

#### 3.1.7. Chestnut Honey

Pollen grains from *Castanea* sp. have sizes ranging between 10 and 25 µm and show a slightly elongated shape, with a circular to slightly elliptical outline in equatorial view [20].

**Figure 8:**
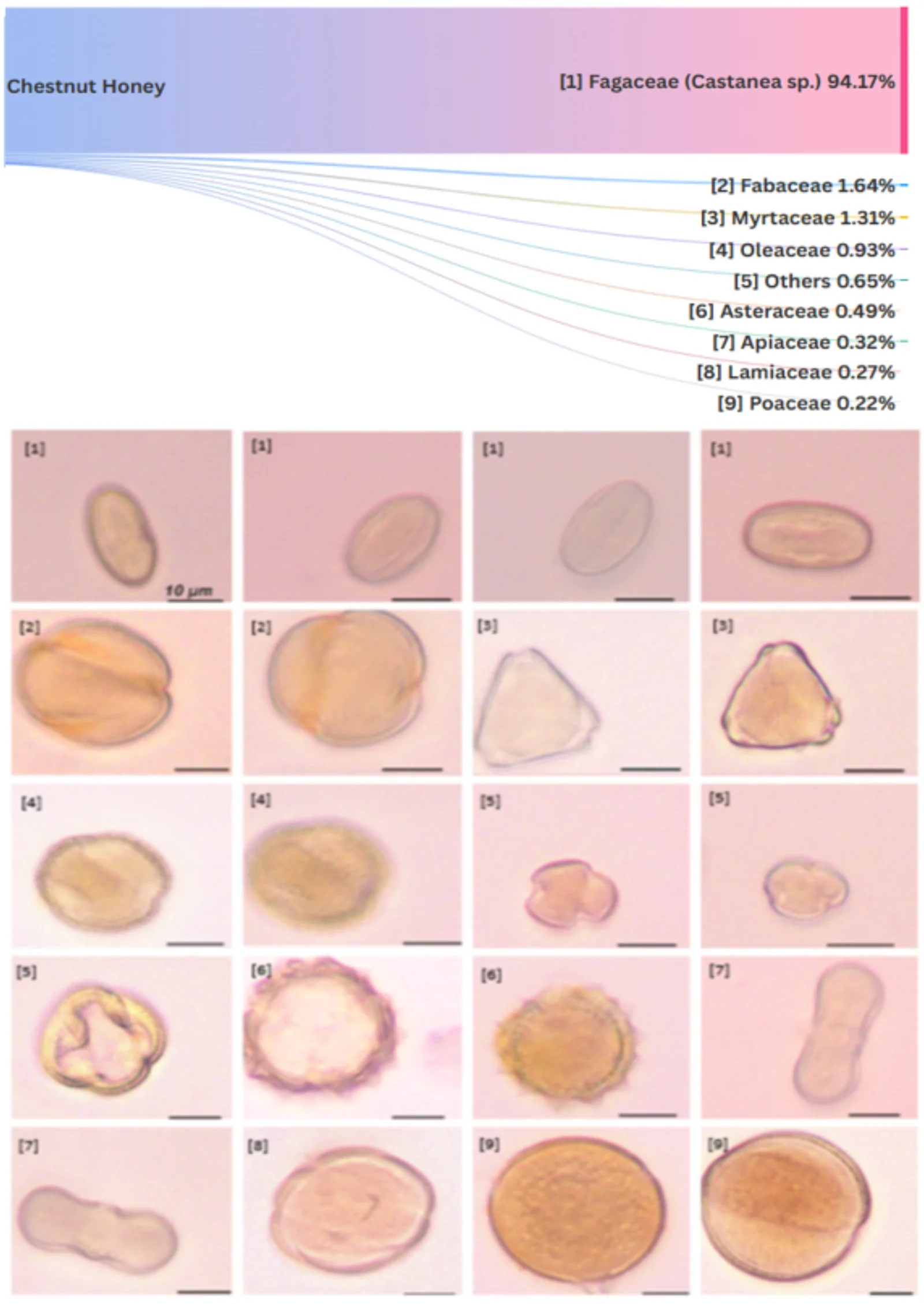
Pollen grains of chestnut honey: [1]–[9].

In the chestnut honey sample, Fagaceae pollen accounted for 94.17% of the total pollen grains (Figure 8). In addition, the sample also contained pollen from plants of other botanical families, including Fabaceae (1.64%), Myrtaceae (1.31%), Oleaceae (0.93%), as well as minor contribution from Asteraceae (0.49%).

#### 3.1.8. Manuka Honey

The pollen of *Leptospermum scoparium*, a species belonging to the Myrtaceae family, is characterized by very small dimensions, less than 20 µm. In polar view, it has a triangular outline with three apertures in the form of furrows (colpi). In equatorial view, it exhibits a flattened oval (oblate) shape. The exine is thin with a smooth or slightly rough surface, and the intine is thickened beneath the apertures [22,23,24].

**Figure 9:**
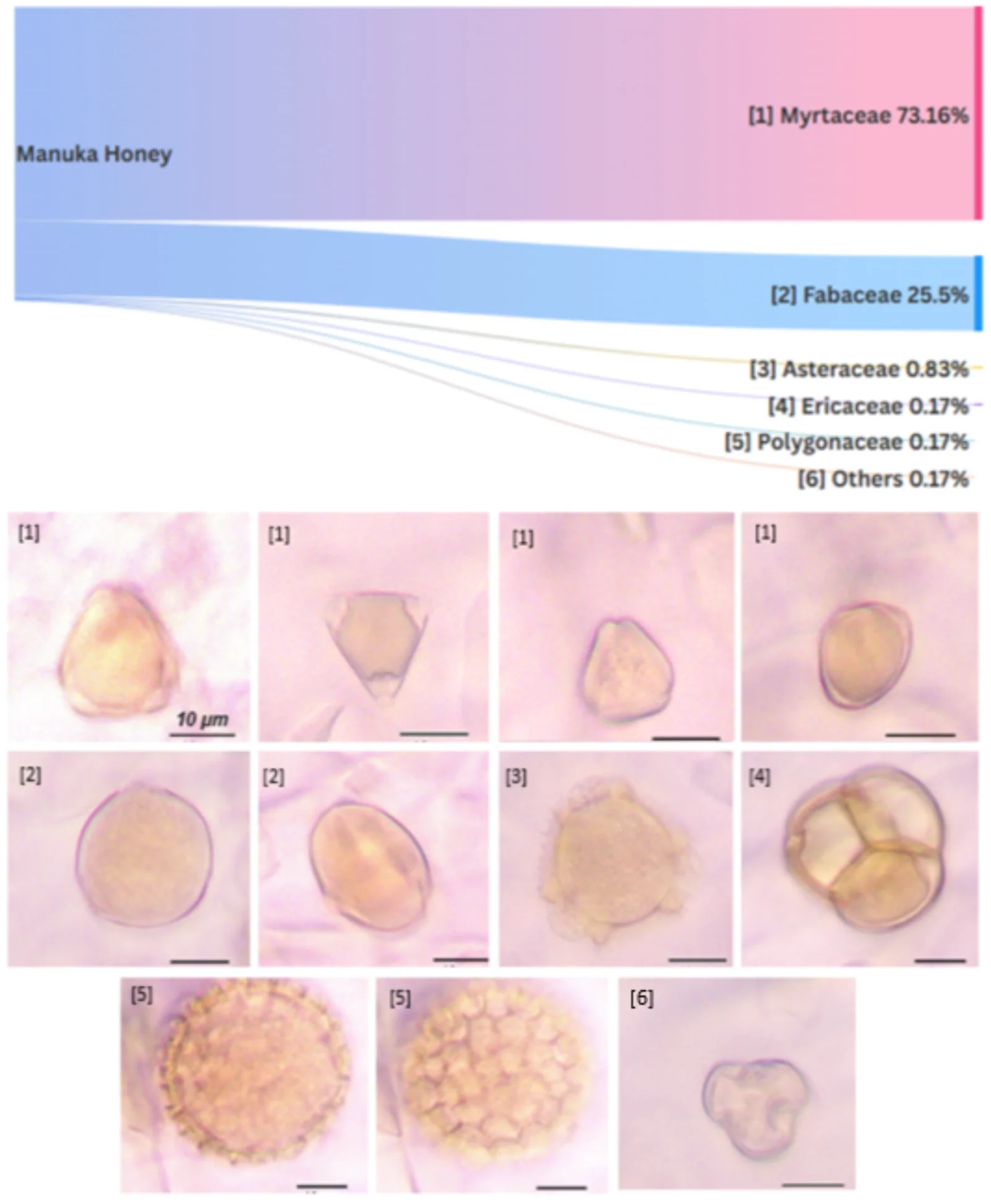
Pollen grains of Manuka honey: [1]–[6].

In the Manuka honey sample, Myrtaceae pollen (*Leptospermum scoparium*) accounted for 73.16% of the total pollen grains (Figure 9). Other identified families included Fabaceae (25.5%), Asteraceae (0.83%), Ericaceae (0.17%), Polygonaceae (0.17%), and other unidentified pollen (0.17%).

#### 3.1.9. Tualang Honey

The presence of pollen grains derived from several botanical taxa in Tualang honey confirms its classification as multifloral rather than monofloral. Among the most interesting are the pollen grains from the Arecaceae family (*Elaeis* sp.) and Fabaceae (*Acacia* sp.). In the analysis of honey samples, the pollen from *Elaeis* sp. appears as a triangular-rounded shape in polar view, with three colpi and a smooth exine. Meanwhile, the pollen from *Acacia* sp. appears as multicellular polyads with a honeycomb-type structure, composed of numerous overlapping pollen units [25].

**Figure 10:**
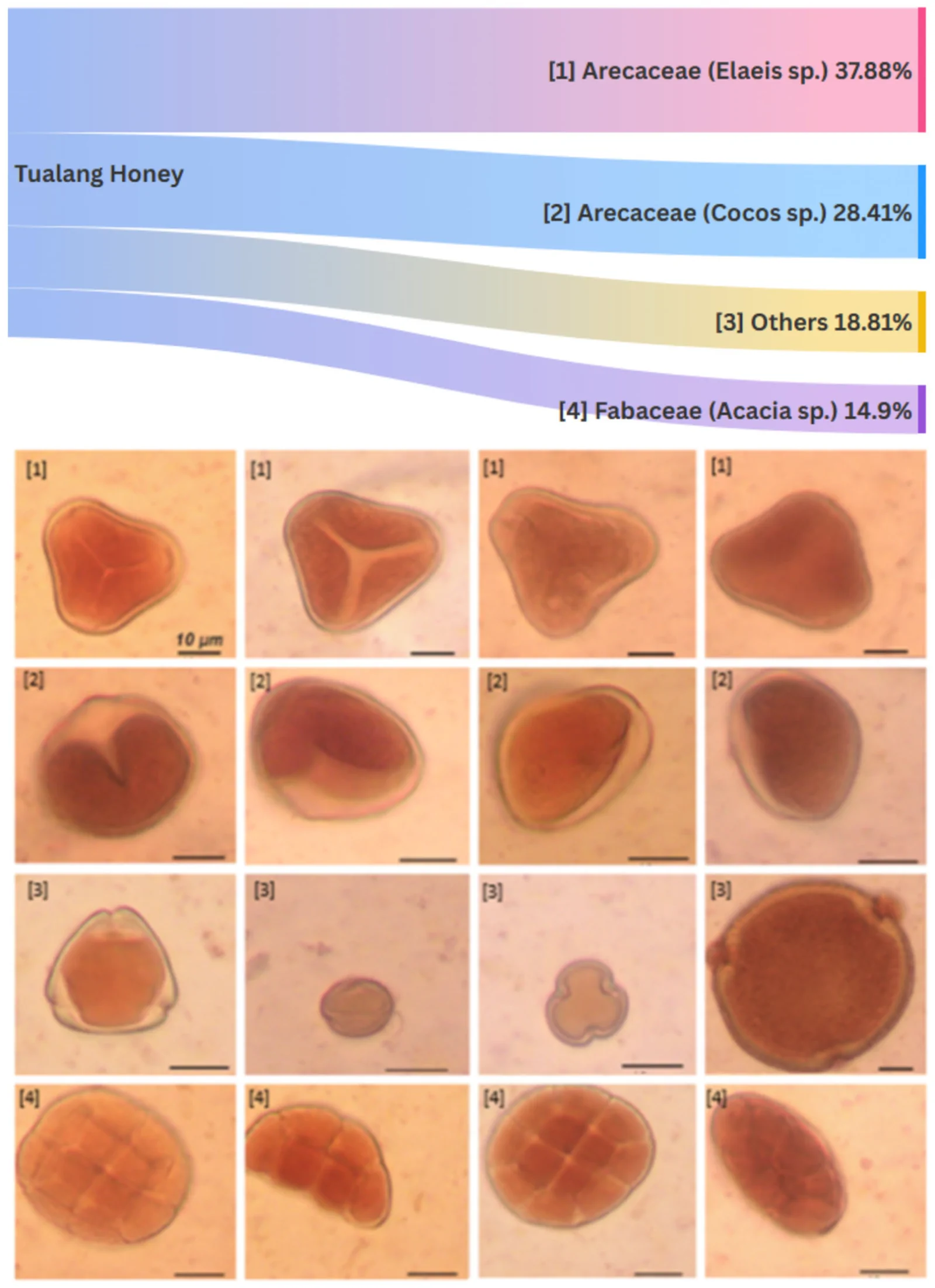
Pollen grains of Tualang honey: [1]–[4].

Pollen analysis conducted on Tualang honey revealed a multifloral character without the dominance of a specific type of pollen. These results confirm a pollen composition typical of Malaysian tropical regions. The pollen species found in the highest proportions were *Elaeis* sp. (37.88%) and *Cocos* sp. (28.41%). A considerable percentage was also reported for pollen derived from plants of the Fabaceae family (*Acacia* sp.), which constituted 14.9% (Figure 10). The “Others” category includes pollen types for which the family origin could not be precisely determined based on microscopic analysis. These may originate from plants belonging to various botanical taxa such as *Ixora* (Rubiaceae), *Melaleuca cajuputi* (Myrtaceae), and *Garcinia* (Clusiaceae) [26].

#### 3.1.10. Multifloral Honey

**Figure 11:**
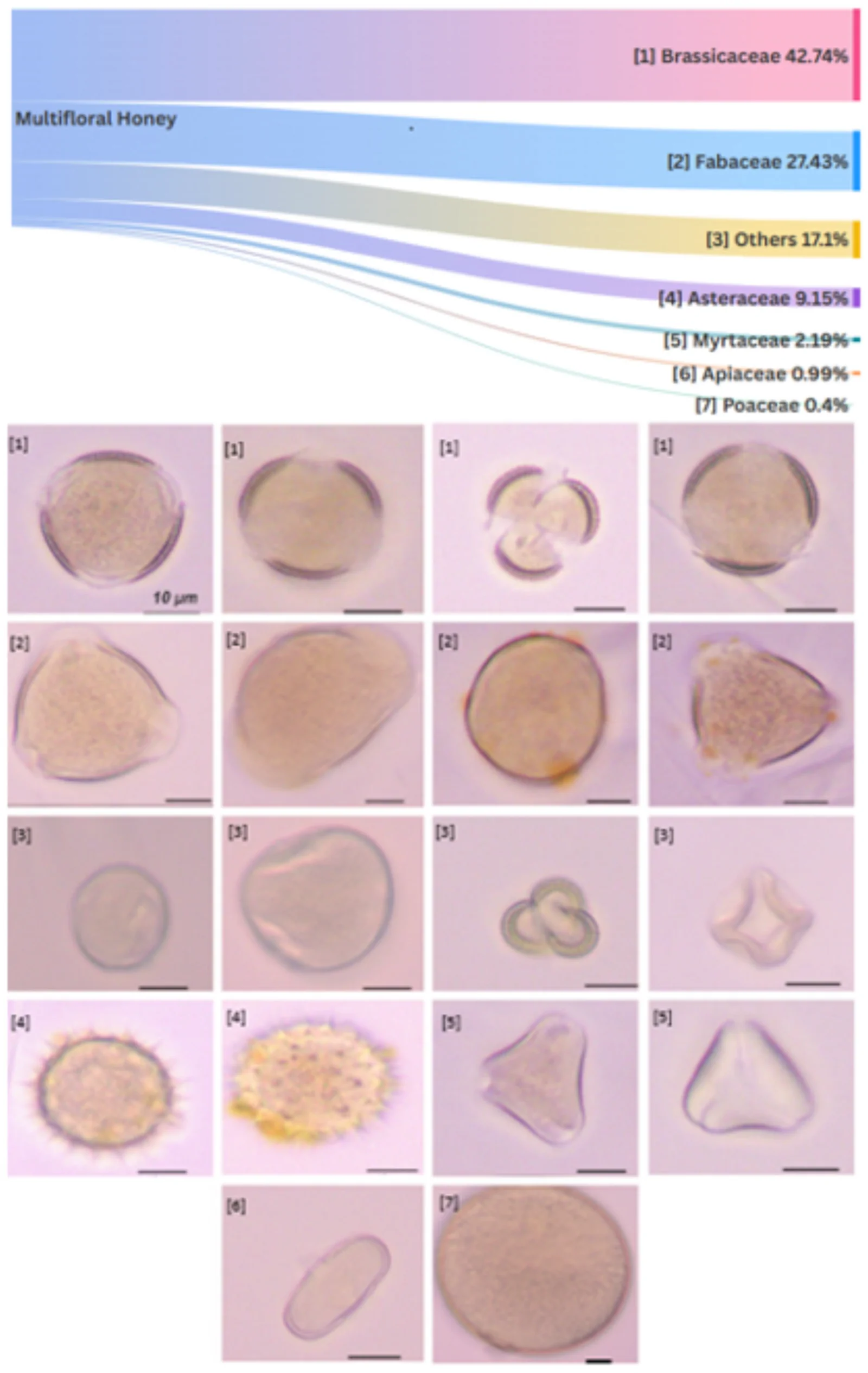
Pollen grains of multifloral honey: [1]–[7].

In the multifloral honey sample, six botanical families were identified. To simplify the analysis, based on grain content percentage, pollen can be classified into four categories: predominant (>45%), secondary (16–45%), important minor (3–15%), and minor (<3%) [27]. In the multifloral honey sample, Brassicaceae represented 42.74%, followed by Fabaceae (27.43%) and Asteraceae (9.15%). Lower percentages were recorded for Myrtaceae (2.19%), Apiaceae (0.99%), and Poaceae (0.4%) (Figure 11).

Findings from a different study conducted in Romania are consistent with the results of the present research, identifying through melissopalynological analysis the most frequent families as: Brassicaceae, Fabaceae, and Asteraceae. A major contributor in many Romanian samples is rapeseed, due to intensive agriculture. It should also be noted that the pollen profile varies according to season and region [28].

#### 3.1.11. Pasture Honey

**Figure 12:**
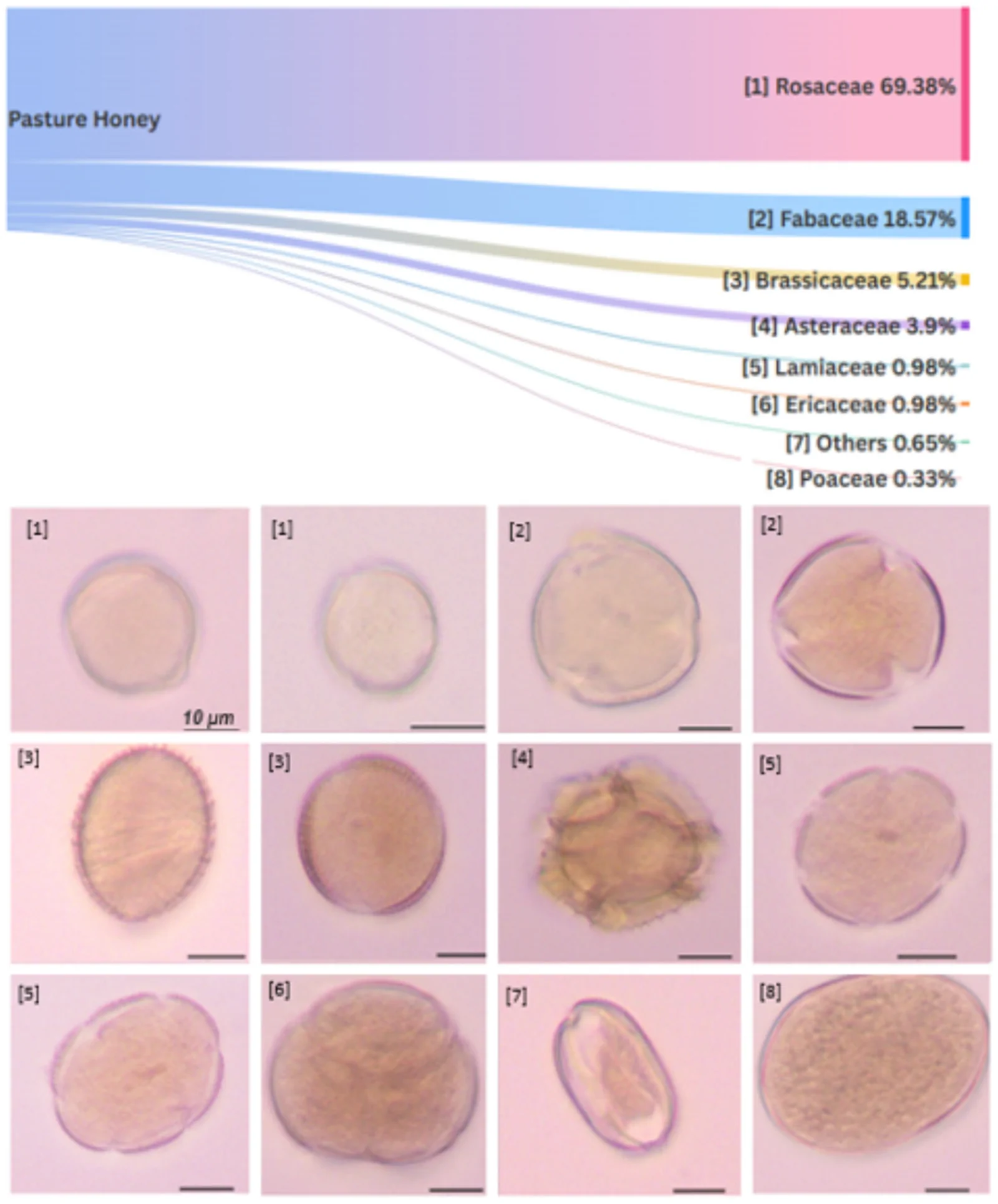
Pollen grains of pasture honey: [1]–[8].

The results of the pollen composition analysis indicate a predominance of the Rosaceae family (69.38%), followed by Fabaceae (18.57%). Smaller amounts of pollen grains from the Brassicaceae (5.21%) and Asteraceae (3.9%) families were also identified. On the opposite end, families such as Lamiaceae (0.98%), Ericaceae (0.98%), Poaceae (0.33%), and the category “Others” (0.65%) (Figure 12) are represented by less than 1%. These results are in line with the specialized literature, which indicates that the pollen profile of honey collected from pastures reflects a high floral diversity [29].

#### 3.1.12. Manna Honey

**Figure 13:**
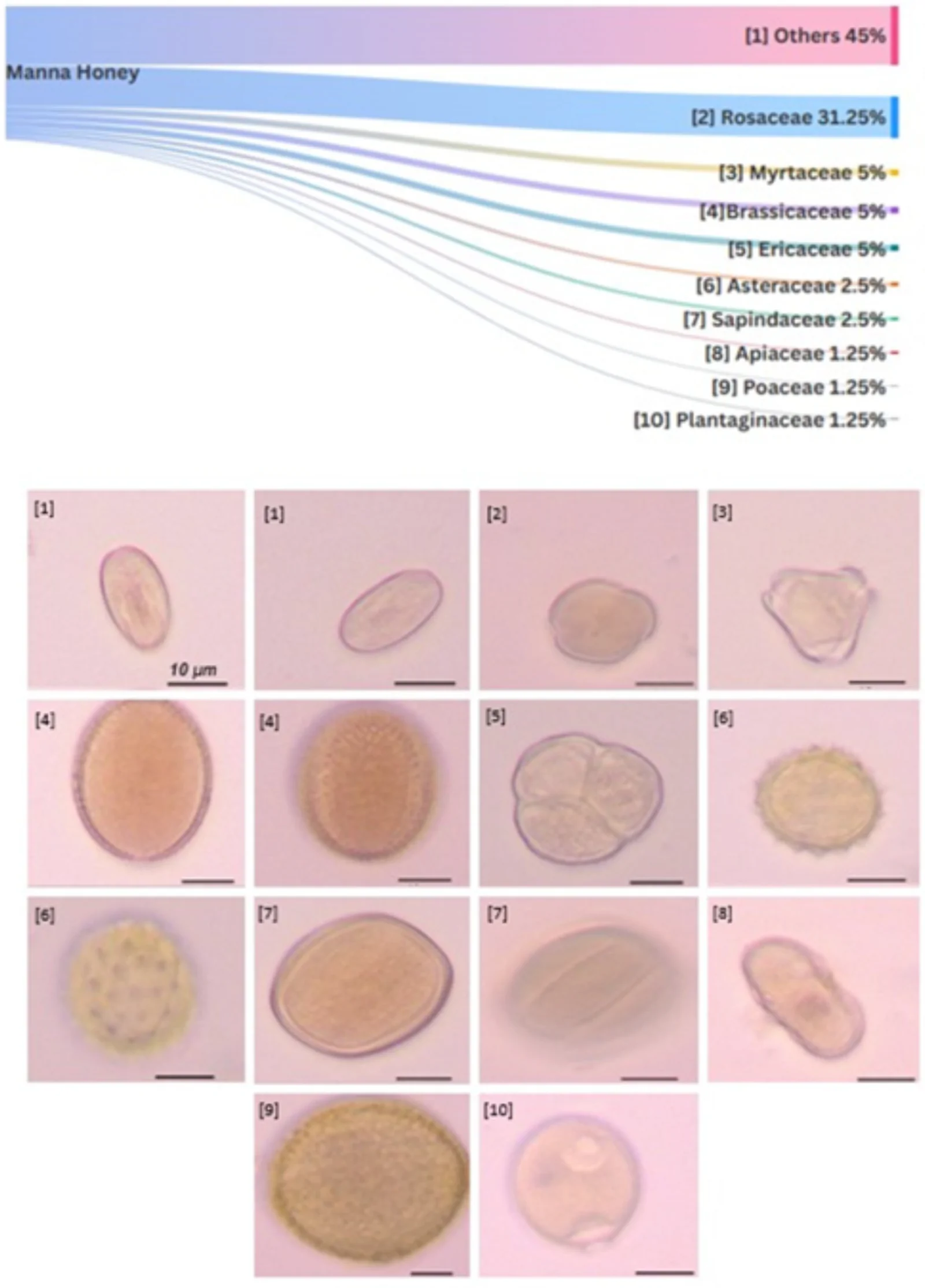
Pollen grains of manna honey: [1]–[10].

The analysis of the manna honey sample revealed a presence of a diverse pollen spectrum commonly found in multifloral honeys. The family identified with a significant pollen contribution is Rosaceae (31.25%), while another category, “Others” (45%), although substantial, could not be precisely identified due to weak differentiating characteristics. In Figure 13, these pollen grains generally have oval or rounded shapes, with smooth or slightly ornamented exines. Such a percentage is acceptable because manna honey is characterized by a predominantly secondary and incidental pollen contribution, coming from forest and background vegetation. This is a trait of manna honey, which does not have a dominant plant source but rather a heterogeneous input from the surrounding vegetation. Other botanical families identified in the pollen spectrum include Myrtaceae (5%), Brassicaceae (5%), Ericaceae (5%). Smaller percentages, ranging from 1.25 to 2.5%, belong to pollen derived from plants of the Asteraceae, Sapindaceae, Apiaceae, Plantaginaceae, and Poaceae families.

### 3.2. Physicochemical Profile of Honey Samples from Different Botanical Origins

As shown in Table 2, the physicochemical parameters of the analyzed honey samples fall within the limits established by international standards (Codex Alimentarius, 2001; European Commission, 2001) [30,31], supporting their quality and authenticity. However, significantly higher HMF values were obtained in two honey samples, Tualang and pasture.

## 4. Discussion

### 4.1. General Discussion

Pollen types morphologically assigned to the Asteraceae family were detected in nine out of 12 analyzed honey samples. As pollen types are defined based on morphological features, they may represent more than one species or genus within this family). The moderate levels of this family may originate either from primary enrichment (bee collection) or from alternative enrichment coming from the air, extraction, and packaging of honey. The Asteraceae family is very widespread globally across a variety of climate zones. Additionally, it is one of the largest plant families comprising over 25,000 species worldwide [32].

The percentages found in the types of honey analyzed ranged from 0.49% (in chestnut honey) to 18.55% (in mint honey). In linden honey, the frequency of Asteraceae was moderate at 8.07%. Melissopalynological analysis highlighted the strongly monofloral character of linden honey, with 84.45% of pollen originating from linden species. For linden honey, the results obtained are comparable to similar studies that highlight the existence of regional variations in the pollen composition of this type of honey. For example, in the specialized literature, a study from Hungary reported linden honeys with a variation of 5.3% to 66.4%, where three samples did not meet the minimum threshold of 30% set by the national standard [33].

Results more closely aligned with the present analysis come from a study carried out in Bulgaria, where linden pollen values tended to be more homogeneous than in the Hungarian samples, suggesting a clearer floral predominance, with few outliers. Linden pollen levels were highest in the specimens collected from the north-central regions of Bulgaria, with values of 78%, 77%, and 75%, respectively [34].

Among the 12 types of honey analyzed, rapeseed honey has the most pronounced botanical specificity. The percentage of pollen originating from *Brassicaceae* (96.86%) in Romanian samples was very high and close to the maximum values reported for Croatian honeys (60.7–99.2%), indicating a very well-defined monofloral character [35].

In the Croatia region, the melissopalynological analysis was conducted on black locust honey and showed that the percentage of specific pollen ranged between 38 and 71% in the first season and 33–66% in the second season, thus confirming that the honey corresponds to the type declared by the producer. These values confirm the authenticity of Croatian black locust honey [36]. Comparable results were obtained in the present study, where black locust pollen accounted for 83.26% of the total pollen.

For the mint honey and thyme honey, the pollen spectrum specific to the source species was slightly over 50%. Compared with other data in the literature, *Mentha* pollen reaches up to 49%, accompanied by Asteraceae and Brassicaceae pollen grains, showing a floral fingerprint quite similar to that of the sample from this analysis [37]. In other studies on monofloral honeys from Romania, it has been reported that the specific pollen for mint honey ranges between 60 and 70% [38]. The percentage of mint pollen in the present analysis, 51.1%, places the values at the lower end of the range, but the monofloral character of the honey is maintained. Similar results were found in Turkey for thyme honey, where Karataş et al. analyzed *Thymus* pollen in monofloral honeys from Southwestern Anatolia and found variable percentages ranging from 48 to 94% [39].

Similarly, for thyme honey from Greece, Karabournioti et al. reported *Thymus* pollen ranging between 27 and 95%, with an average value of approximately 62% [40].

Chestnut honey exhibits a strong monofloral character, with a chestnut pollen percentage that exceeds the threshold required for classifying honey as monofloral. Melissopalynological analysis confirms the floral homogeneity of the nectar-producing plant source, a characteristic feature of this type of honey. The pollen component obtained (94.17% for current study) is consistent with the data reported in other international studies, which confirm that chestnut honey produces large quantities of pollen, found in high concentrations in honey. A study from Turkey shows that in chestnut honey species-specific pollen appears in the range of 77.2% to 98.5% [41]. Data from the specialized literature confirm that chestnut honey contains high concentrations of pollen, frequently exceeding 70–80%. For chestnut honey, international codes indicate that the rate of the dominant pollen should be >70% [42].

The results of the present melissopalynological analysis of Manuka honey showed the dominance of characteristic pollen, representing 73.16% of the total pollen content, indicating its botanical origin. According to Moar et al., Manuka honey is typically characterized by a high proportion of pollen grains from *Leptospermum scoparium*, with minimum pollen frequencies of approximately 70%. Moar’s study also indicates the frequent presence of pollen grains from the Fabaceae family (especially white clover) in many New Zealand honey samples, including Manuka honey, where it most often occurs as secondary pollen [42]. The present study confirms this finding, with *Fabaceae* pollen accounting for approximately 25% of the total pollen content. Thus, the pollen profile of the Manuka honey analyzed in the present study closely matches the “classic” profile described by Moar for confirming botanical authenticity [43].

Tualang honey exhibits several particular features, such as being produced by *Apis dorsata*, which places its hives in the high branches of a tree species that occurs mainly in tropical rainforest habitats, the Tualang tree (*Koompassia excelsa*). The tree can reach heights of over 50 m. According to Hamid et al., Tualang honey is a multifloral honey [26]. Among the types of pollen analyzed, particular importance is given to pollen grains from the Arecaceae family, mainly represented by the species *Elaeis* sp. and *Cocos* sp. These two identified species constitute frequent and consistent plant sources of pollen for the bee *Apis dorsata* in the Malaysian region. This aspect indicates the tropical origin of these plants and emphasizes their essential role in characterizing the palynological profile typical of Tualang honey [26,44]. Species such as *Ixora* (Rubiaceae), *Melaleuca cajuputi* (Myrtaceae), and *Garcinia* (Clusiaceae) can be classified under the “Others” category in this study, although they cannot be clearly identified. Nevertheless, the three common dominant species—*Elaeis* sp., *Cocos* sp., and *Acacia* sp.—demonstrate the multifloral nature of Tualang honey [26].

Although manna honey does not originate from flower nectar but rather from insect (aphid) secretions, variable amounts of pollen may accidentally enter. Therefore, the pollen found in the analysis occurs incidentally and can be highly diverse. According to data from a study analyzing pollen in manna, the following pollen spectrum was identified: Sapindaceae (29.11–30.11%), Fagaceae (28.67–29.99%), Rosaceae (21.55–28.78%), Asteraceae (22.21–28.76%) [45]. Our analysis found several important families in common with those of this previous report: Rosaceae, Asteraceae, Sapindaceae. It is possible that other types of pollen may also be common, for example from the Fagaceae family; these can be classified under the category ‘Others’ because they could not be precisely identified. In manna honey, pollen content may not represent the primary criterion for classification, as its production is associated with plant exudates rather than floral nectar.

In all types of honey analyzed, the pollen spectrum belonging to the Poaceae family was identified in low concentrations, showing a relatively homogeneous distribution among the samples. Poaceae is frequently found in honey samples as a result of passive contamination from atmospheric particles; it represents an anemophilous pollen type, mainly originating from agricultural areas where flowering crops produce large quantities of wind-dispersed pollen [46]. In the present study, pollen grains from the Poaceae family were detected in about one of every two analyzed honey samples, with a frequency of 0.21% for rapeseed honey and a maximum of 1.57% for linden honey.

### 4.2. Comparative Melissopalynological Profiles by Climatic Region

**Figure 14:**
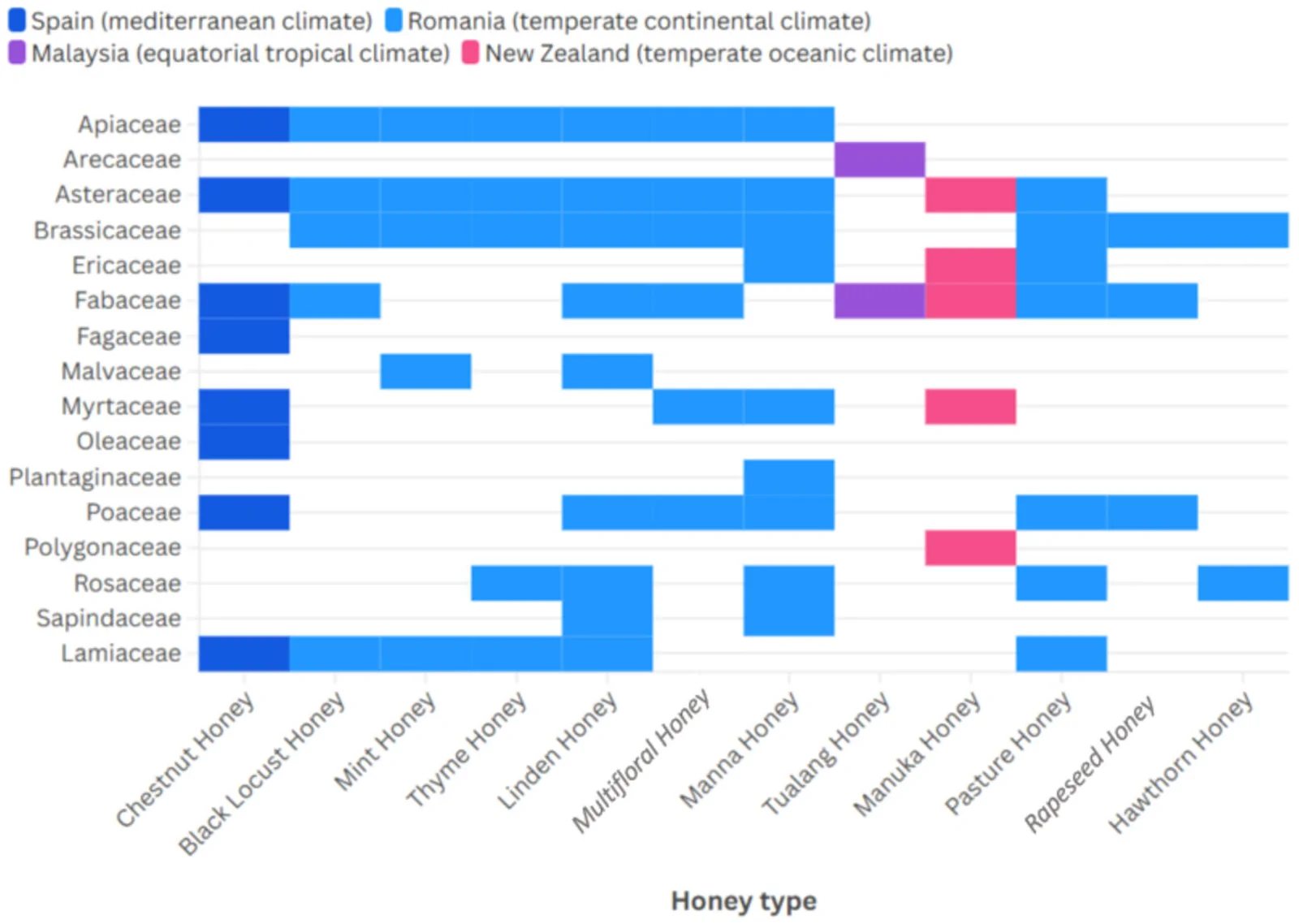
Botanical families identified in the pollen of different honey types in relation to Mediterranean, temperate continental, tropical equatorial and temperate oceanic climates.

A comparison of different climatic zones shows that the distribution of pollen originating from various botanical families depends on the characteristic vegetation of each climate. In addition to the influence of the local flora, the climate also has an impact on the pollen composition of honey, although climate is also directly impacting the flora, and thus its effects cannot be easily disentangled from those of flora (Figure 14).

The study of honey samples collected from Romania, Spain, Malaysia, and New Zealand outlined a distinct pollen profile correlated both with the local flora and with the bee species involved in honey production. Chestnut honey from Spain was dominated by pollen from the Fagaceae family, alongside Apiaceae and Asteraceae, which are characteristic of Mediterranean climate areas. This pollen structure is specific to honey produced by *Apis mellifera*, the species used in Spanish beekeeping.

In melissopalynological analysis, for the honey samples from Romania (n = 9), a high pollen diversity specific to the region with mixed botanical origin was observed. The main families, both in frequency and percentage, are Fabaceae, Brassicaceae, Rosaceae, and Asteraceae. The samples originating from Romania, which belong to the temperate continental climate, exhibited a high pollen diversity, characteristic of areas with mixed plain and hill flora. This variability is typical of the types of honey produced by *Apis mellifera*, a species found throughout Europe and known for its capacity to make use of wide spectrum of plant species.

The pollen profile of Tualang honey originates from the equatorial tropical rainforest of Malaysia [47]. The identification of pollen from the Arecaceae (*Elaeis* sp., *Cocos* sp.) and Fabaceae (tropical *Acacia* species) families confirms the geographical origin of the humid tropical forests. This pollen profile is characteristic of honey produced by *Apis dorsata*, the Asian giant honeybee, a wild species that builds its hives in large trees such as *Koompassia excelsa* (known as Tualang) [26].

For Manuka honey, the study highlighted the presence of pollen from the Myrtaceae family, especially *Leptospermum scoparium*. In addition, pollen from families such as Ericaceae and Asteraceae, common in the oceanic temperate flora of New Zealand, was also identified. For Europe and New Zealand, the pollen footprint is associated with *Apis mellifera*, whereas in Malaysia, the pollen profile is associated with *Apis dorsata*, the giant bee characteristic of wild tropical honeys. These results indicate that through melissopalynological analysis, the geographic origin of honey can be differentiated. Figure 14 illustrates the distribution of botanical families identified in the pollen of different honey types across climatic regions, highlighting regional differences in pollen composition. 

### 4.3. Floral Diversity and Dominance Among Honey Types

This study showed that honeys labeled and confirmed through melissopalynological analysis as monofloral are predominantly characterized by the botanical families of their pollen-source plants. Rapeseed, chestnut, linden and black locust honeys showed a strong predominance of the pollen of the respective species. Honeys derived from aromatic plants, particularly from the Lamiaceae family, exhibited borderline dominance, while multifloral, pasture, and manna honeys highlight a greater diversity in botanical families of pollen-source plants, including Brassicaceae, Rosaceae, Fabaceae, and Asteraceae.

For Manuka honey, the predominance of Myrtaceae pollen confirms its botanical origin from *Leptospermum scoparium* in New Zealand. This perfectly illustrates how melissopalynological analysis can differentiate authentic Manuka honey from substitutes or adulterations.

Tualang honey is multifloral, with family characteristics typical of the tropical climate, particularly Arecaceae and Fabaceae.

Table 3 clearly shows how, through melissopalynological analysis, specific patterns for each region can be outlined. The presence of numerous pollen grains derived from a variety of plant sources confirms the authenticity of the honey, as well as the diverse flowering plants utilized by the bees.

### 4.4. Physicochemical Parameters Relevant to Honey Quality and Authenticity

The moisture content of all samples was below the maximum accepted threshold (20%), indicating proper maturation and storage conditions [48]. The content of reducing sugars was consistent with typical values reported for natural honeys, while the apparent sucrose levels were low, suggesting the absence of adulteration or premature harvesting [49]. Diastase activity values also confirmed the samples freshness and enzymatic integrity, with all values within acceptable ranges [50].

In addition, the HMF content, an important indicator of heat exposure and storage conditions, was within the recommended limits, indicating minimal thermal degradation, except for Tualang and pasture honey. The markedly elevated HMF values observed in these honey samples suggest possible thermal exposure or prolonged storage, as HMF accumulates during heating and aging. In the case of Tualang honey, its tropical origin may contribute to accelerated HMF formation due to higher ambient temperatures. However, the measured value exceeds even the limits accepted for tropical honeys, suggesting the influence of additional post-harvest factors. For pasture honey, the elevated HMF content is most likely associated with storage conditions or processing history, as HMF formation is not related to botanical origin but rather to storage temperature and duration. These results indicate that HMF reflects post-harvest conditions rather than the botanical origin assessed through melissopalynological analysis, as previously reported in the literature [2].

A limitation of this study is the unequal sample sizes across geographic regions, particularly for non-European honeys. However, for the honey samples from Romania, the geographic origin was confirmed through direct communication with local beekeepers. Additionally, although melissopalynological analysis was used to assess the botanical origin, the geographic origin of non-local samples could not be fully controlled as it relied on the information available on the sample labels. Future studies should aim to include larger sets of samples and a more comprehensive analytical approach.

## 5. Conclusions

This study demonstrated that melissopalynological analysis represents a reliable tool for botanical authentication of honey. Monofloral honeys, such as rapeseed, linden, chestnut, and black locust, exhibited a clear predominance of their characteristic pollen types, exceeding the established 45% threshold required for monofloral classification. In contrast, thyme and mint honeys showed moderate predominance consistent with borderline monofloral classification.

Physicochemical parameters were generally in line with international quality standards, supporting the authenticity and proper handling of most samples. However, elevated HMF values obtained in Tualang and pasture honeys highlighted the influence of post-harvest factors (such as storage and thermal exposure) on honey quality.

Overall, the integration of melissopalynological and physicochemical analyses provides a complementary approach for honey characterization, allowing differentiation between botanical origin and quality-related parameters.

## Figures and Tables

**Table 1:** Geographical origin and source of the analyzed honey samples.

Honey Type	Geographical Origin	Coordinates	Source
Linden (*Tilia* sp.)	Snagov, Romania	44.71° N, 26.15° E	Beekeeper
Rapeseed (*Brassica* sp.)	Giurgiu, Romania	43.90° N, 25.97° E	Beekeeper
Black locust (*Robinia* sp.)	Călărași, Romania	44.20° N, 27.33° E	Beekeeper
Hawthorn (*Crataegus* sp.)	Argeș, Romania	44.90° N, 24.73° E	Beekeeper
Mint (*Mentha* sp.)	Constanța, Romania	44.18° N, 28.65° E	Beekeeper
Thyme (*Thymus* sp.)	Dolj, Romania	44.32° N, 23.80° E	Beekeeper
Chestnut (*Castanea* sp.)	Spain	Not available	Commercial
Manuka (*Leptospermum* sp.)	New Zealand	Not available	Commercial
Tualang (*Koompassia* sp.)	Malaysia	Not available	Commercial
Multifloral (mixed floral sources)	Dolj, Romania	44.32° N, 23.80° E	Beekeeper
Pasture (mixed floral sources)	Argeș, Romania	44.90° N, 24.73° E	Beekeeper
Manna (forest honeydew origin)	Dâmbovița, Romania	44.77° N, 25.32° E	Beekeeper

**Table 2:** Physicochemical characteristics of the analyzed honey samples.

Honey Type	Water (%)	Sucrose (%)	Reducing Sugars (%)	Diastase Index	HMF (mg/100 g)
Linden	15.20 ± 0.30	3.33 ± 0.22	73.27 ± 4.67	17.90 ± 2.28	0.75 ± 0.09
Rapeseed	15.80 ± 0.29	2.20 ± 0.14	78.00 ± 4.98	17.90 ± 2.28	2.38 ± 0.30
Black locust	17.20 ± 0.32	3.54 ± 0.23	65.97 ± 4.71	17.90 ± 2.28	3.42 ± 0.43
Hawthorn	16.00 ± 0.29	2.02 ± 0.13	74.50 ± 4.75	23.80 ± 3.03	1.23 ± 0.15
Mint	16.00 ± 0.29	1.74 ± 0.11	77.27 ± 4.93	17.90 ± 2.28	8.51 ± 1.07
Thyme	16.00 ± 0.29	2.82 ± 0.19	74.53 ± 4.76	10.90 ± 1.39	4.07 ± 0.51
Chestnut	16.60 ± 0.31	9.12 ± 0.60	65.97 ± 4.21	29.40 ± 3.75	2.28 ± 0.29
Manuka	15.20 ± 0.30	3.26 ± 0.21	75.17 ± 4.80	17.90 ± 2.28	6.01 ± 0.76
Tualang	15.00 ± 0.28	4.58 ± 0.30	72.18 ± 4.61	10.90 ± 1.39	43.85 ± 5.52
Multifloral	15.60 ± 0.29	1.87 ± 0.12	77.13 ± 4.92	17.90 ± 2.28	8.26 ± 1.04
Pasture	16.40 ± 0.30	2.35 ± 0.15	75.63 ± 4.83	17.90 ± 2.28	28.36 ± 3.57
Manna	16.40 ± 0.30	9.82 ± 0.65	64.93 ± 4.14	38.50 ± 4.90	1.67 ± 0.21

**Table 3:** Distribution (%) of pollen across botanical families in the analyzed honey types.

	Frequency (%)																
Honey Type	Malvaceae	Asteraceae	Rosaceae	Fabaceae	Fagaceae	Brassicaceae	Lamiaceae	Myrtaceae	Apiaceae	Sapindaceae	Ericaceae	Polygonaceae	Oleaceae	Plantaginaceae	Poaceae	Arecaceae	Others
Linden	84.45	8.07	2.37	0.78	-	0.78	0.4	-	0.4	0.4	-	-	-	-	1.57	-	0.78
Rapeseed	-	-	-	2.15	-	96.86	-	-	-	-	-	-	-	-	0.21	-	0.78
Black locust	-	1.19	-	83.26	-	9.56	0.2	-	2.2	-	-	-	-	-	-	-	3.59
Hawthorn	-	-	55.55	-	-	8.75	-	-	-	-	-	-	-	-	-	-	35.7
Mint	0.17	18.55	-	-	-	12.14	51.1	-	2.53	-	-	-	-	-	-	-	15.51
Thyme	-	6.26	12.72	-	-	27.4	52.84	-	0.78	-	-	-	-	-	-	-	-
Chestnut	-	0.49	-	1.64	94.17	-	0.27	1.31	0.32	-	-	-	0.93	-	0.22	-	0.65
Manuka	-	0.83	-	25.5	-	-	-	73.16	-	-	0.17	0.17	-	-	-	-	0.17
Tualang	-	-	-	14.9	-	-	-	-	-	-	-	-	-	-	-	66.29	18.81
Multifloral	-	9.15	-	27.43	-	42.74	-	2.19	0.99	-	-	-	-	-	0.4	-	17.1
Pasture	-	3.9	69.38	18.57	-	5.21	0.98	-	-	-	0.98	-	-	-	0.33	-	0.65
Manna	-	2.5	31.25	-	-	5.0	-	5.0	1.25	2.5	5.0	-	-	1.25	1.25	-	45.0

## Data Availability

The original contributions presented in this study are included in the article. Further inquiries can be directed to the corresponding author.
